# The Times They Are A-Changin’: Heterochrony in Plant Development and Evolution

**DOI:** 10.3389/fpls.2018.01349

**Published:** 2018-09-18

**Authors:** Manuel Buendía-Monreal, C. Stewart Gillmor

**Affiliations:** Laboratorio Nacional de Genómica para la Biodiversidad (Langebio), Unidad de Genómica Avanzada, Centro de Investigación y de Estudios Avanzados del Instituto Politécnico Nacional (CINVESTAV-IPN), Guanajuato, Mexico

**Keywords:** heterochrony, developmental timing, plant development, plant evolution, cell cycle, miR156

## Abstract

Alterations in the timing of developmental programs during evolution, that lead to changes in the shape, or size of organs, are known as heterochrony. Heterochrony has been widely studied in animals, but has often been neglected in plants. During plant evolution, heterochronic shifts have played a key role in the origin and diversification of leaves, roots, flowers, and fruits. Heterochrony that results in a juvenile or simpler outcome is known as paedomorphosis, while an adult or more complex outcome is called peramorphosis. Mechanisms that alter developmental timing at the cellular level affect cell proliferation or differentiation, while those acting at the tissue or organismal level change endogenous aging pathways, morphogen signaling, and metabolism. We believe that wider consideration of heterochrony in the context of evolution will contribute to a better understanding of plant development.

## The Different Types of Heterochrony

In the 1870s, Ernst Haeckel identified temporal and spatial changes in development in a descendant relative to its ancestor as the two mechanisms most important for evolution (Haeckel, 1875). Haeckel named spatial changes heterotopy, and temporal changes heterochrony.

However, the meaning of heterochrony has changed since Haeckel first coined the term. Haeckel used the term heterochrony to refer to deviations from his well-known “Biogenetic Law,” which states that the sequence of developmental events (ontogeny) largely recapitulates the sequence of events in the evolutionary history of the species (phylogeny) (Haeckel, 1875). Thus, heterochrony originally referred to a change in the timing of appearance of a feature in a developmental sequence of an organism, relative to the sequence that occurred in the organism’s phylogeny. In the middle of the 20th century, De Beer (1951) uncoupled heterochrony from recapitulation. He used heterochrony to denote differences in the timing of developmental events when comparing two related species, to explain how heterochrony could generate diversity among organisms. Gould (1977) re-associated the concept of heterochrony to recapitulation, defining heterochrony as “changes in the relative time of appearance and rate of development for characters already present in ancestors,” emphasizing changes in relative size and shape, rather than in the timing of developmental events, to detect heterochrony. At the turn of the 21st century, Smith proposed that it would be more useful to define two types of heterochrony: ‘growth heterochrony,’ which emphasizes changes in final size and shape; and ‘sequence heterochrony,’ which is closer to the original usage of Haeckel and de Beer, and allows explanation of phenotypic variation by changes in the timing of developmental events (Smith, 2002, 2003; Keyte and Smith, 2014).

**FIGURE 1:**
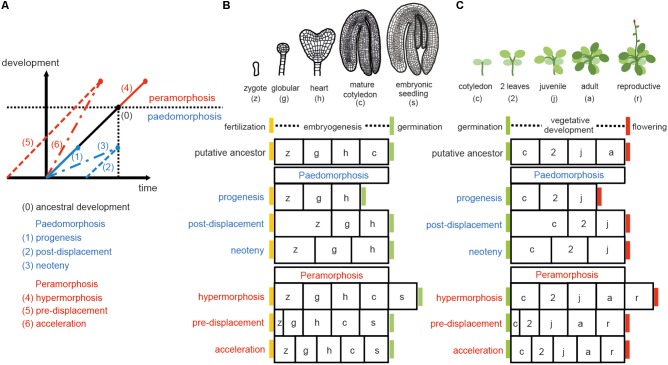
Types of heterochrony. **(A)** Schematic representation of the 6 types of heterochrony. The black line represents the time required to reach a certain developmental stage in the ancestral ontogeny. Blue lines show the 3 types of paedomorphosis: progenesis (precocious offset), post-displacement (delayed onset) and neoteny (slower developmental rate). Red lines show the 3 types of peramorphosis: hypermorphosis (delayed offset), pre-displacement (precocious onset), and acceleration (higher developmental rate). Drawing in **(A)** is based on Alberch et al. (1979) and Geuten and Coenen (2013). **(B)** Heterochrony scenarios for embryogenesis. Arabidopsis embryogenesis is taken here as the hypothetical ancestral development, divided in four stages for illustrative purposes. A fifth stage, where the embryo has produced the first two leaves plus the two cotyledons (denoted embryonic seedling), is proposed as the final stage in peramorphic embryogenesis, whereas paedomorphic embryogenesis is expected to conclude at the heart stage. **(C)** Heterochrony scenarios for vegetative development. In this case, Arabidopsis vegetative development, where the plant continues producing cauline/reproductive leaves on the stem before flowering (drawn on the top), represents a hypothetical case of peramorphic vegetative development, compared to a hypothetical ancestor which flowers at the adult stage without producing cauline leaves. Paedomorphic vegetative development is predicted to result in plants flowering at the juvenile stage.

Sequence heterochrony (hereafter referred to as ‘heterochrony’) can be classified in two categories: paedomorphosis and peramorphosis. When compared to ancestral development, paedomorphosis results in a juvenile or simple outcome, whereas peramorphosis results in an adult or more complex phenotype. Each of these two categories of heterochrony can result from variation in timing of the onset, offset or rate of a developmental process, as proposed by Alberch et al. (1979). This variation can result in 6 different types of heterochrony (**Figure 1**). Paedomorphosis can result from the precocious end of a developmental process (progenesis), from a delayed start of the process (post-displacement), or from a slower rate of development (neoteny). Peramorphosis is the result of an extended period of development due to a later termination (hypermorphosis) or an earlier onset (pre-displacement), or of a higher rate of development (acceleration) (**Figure 1A**). Hypothetical examples for peramorphosis and paedomorphosis in plant embryogenesis and vegetative development are shown in **Figures 1B,C**.

While these classifications are useful to illustrate changes in developmental timing, when considering real life examples, it is often difficult to distinguish between different kinds of heterochrony. In many cases, evolution can result in distinct types of heterochrony, each occurring at a discrete stage of a developmental process (reviewed in plants by Li and Johnston, 2000).

## Heterochrony in the Evolution and Diversification of Plants

Land plants (embryophytes) have undergone many morphological innovations since their emergence in the mid-Ordovician period, about 470 million years ago. Early diverging lineages originated over a period of more than 100 million years, during the Silurian and early Devonian periods (Kenrick and Crane, 1997; Pires and Dolan, 2012; Harrison and Morris, 2018). Embryophytes evolved from a freshwater algae ancestor that was related to the extant charophyte groups Charales, Coleochaetales, and Zygnematales. The transition to growth on land, as observed in bryophytes (hornworts, mosses, and liverworts), involved the origin of spores, alternation of gametophyte and sporophyte generations, uniaxial forms, and three-dimensional growth. Innovations of terrestrial vascular plants (lycophytes, monilophytes, and spermatophytes) included bifurcation, indeterminacy, sporophytic dominance, axillary branching, and the formation of meristems, leaves, and roots (Harrison, 2017).

Several of the evolutionary steps above have been linked to heterochrony. For the origin of sporogenesis, bryophyte data suggest that spores were produced directly from zygotes in a process involving precocious cytokinesis, acceleration of meiosis and delayed wall deposition from the zygote to the meiospores (Brown and Lemmon, 2011). The branched sporophyte (polisporangiophyte) has been hypothesized to have evolved by extended vegetative growth of the apical cell. This longer period of vegetative growth was proposed to result in a prolonged embryonic axis, shoot branching, and a delay in the transition to reproductive growth, producing the sporangium (Rothwell et al., 2014; Tomescu et al., 2014). Additional studies of plant fossils should provide more evidence for ancient plant morphologies, which would allow comparison between contemporary and extinct forms (Rothwell et al., 2014).

In extant plants, heterochronic changes have been identified in gametophyte development, embryogenesis, vegetative development, shoot maturation, and floral morphogenesis. The female gametophyte of *Gnetum* is structurally divergent from other plants because of differences in the timing of fertilization and somatic development. Temporal alterations in cell cycle progression have contributed to diversified temporal patterns of spermatogenesis and gamete fusion during fertilization (Friedman, 1999; Tian et al., 2005). In a case of progenesis, fertilization occurs at a free nuclear stage of somatic development, a juvenile stage compared to the ancestral somatic ontogeny, precluding the differentiation of egg cells (Friedman and Carmichael, 1998). The apomictic development of *Boechera* ovules has been associated with heterochronic gene expression patterns compared to non-apomictic (sexual) ovules (Sharbel et al., 2010). The development of Rafflesiaceae, a holoparasitic plant family which infects grapevines, shows two heterochronic shifts: an arrest at the proembryonic stage, which can be considered an example of neoteny, and acceleration of the transition from the undifferentiated endophyte to flowering, skipping vegetative shoot maturation (Nikolov et al., 2014).

A Quantitative Trait Loci (QTL) analysis comparing *Eucalyptus globulus* populations with precocious vegetative phase change and populations in which vegetative phase change is delayed several years identified the expression of the microRNA EglMIR156.5 as responsible for heterochronic variation in vegetative phase change in *E. globulus* (Hudson et al., 2014). Another QTL analysis concluded that heterochrony underlies natural variation in *Cardamine hirsuta* leaf form (Cartolano et al., 2015). QTL mapping determined that the effect is caused by *cis*-regulatory variation in the floral repressor *ChFLC* such that populations with low-expressing *ChFLC* alleles show both early flowering and accelerated acquisition of adult leaf traits, particularly increased leaflet number. Morphometric and QTL analyses have determined that heterochronic mutations contribute to natural variation in *Antirrhinum* and to grapevine heteroblasty (Costa et al., 2012; Chitwood et al., 2016). A Principal Component Analysis (PCA) of the ontogenetic trajectories of leaf form among the three genera of marsileaceous ferns (*Marsilea*, *Regnellidium*, and *Pilularia*) suggested that they show a paedomorphic phenotype, compared to the more complex ancestral development, caused by accelerated growth rate and early termination at a simplified leaf form (Pryer and Hearn, 2009).

Evolutionary diversity in inflorescence architecture of the Solanaceae is modulated by heterochronic shifts in the acquisition of floral fate (Lippman et al., 2008; Park et al., 2012). A comparison of transcriptomes of meristem maturation from five domesticated and wild Solanaceae species revealed a peak of expression divergence, resembling the “inverse hourglass” model for animal embryogenesis, which states that a mid-development period of divergence drives morphological variation (Lemmon et al., 2016). In grasses, a delay in the shoot meristem (SM) to floral meristem (FM) transition results in more complex panicles (Kyozuka et al., 2014). Poplars (*Populus sp*.) and willows (*Salix sp*.) bear compact unisexual inflorescences known as “catkins,” which have been proposed to be evolved from a simplification of the panicle form by an early SM to FM transition (Cronk et al., 2015). Heterochronic changes have also contributed to natural variation in flowering time and shoot architecture among *Mimulus guttatus* populations (Baker and Diggle, 2011).

Morphological diversity of the perianth in Dipsacoideae is caused by heterochronic changes in organ initiation, specifically in the number of sepals (Naghiloo and Claßen-Bockhoff, 2017). The great shape diversity of sepals among Iris species is due more to heterochrony than to heterotopic changes (Guo, 2015). A study in Brassicaceae showed that evolution of corolla monosymmetry from the polysymmetrical ancestral flower involved a heterochronic shift in the expression of *CYC2* genes (a clade of *TCP* transcription factors) from early adaxial expression in ancestral floral meristems, to a later adaxial expression in petal development (Busch et al., 2012). Heterochronic, but not heterotopic, *CYC2* expression has also been associated with a loss of papillate conical cells in petals and a shift to bird-pollination system in *Lotus* (Ojeda et al., 2017). A paedomorphic morphology, in which the flowers hold mature pollen in unopened bud-like structures, led to specialized pollination in a clade of Madagascar vines (Euphorbiaceae) (Armbruster et al., 2013). The evolution of cleistogamous capitulum from a chasmogamous ancestral state is a classic example of paedomorphosis, since the cleistogamous shape shows juvenile traits (Lord and Hill, 1987). Cleistogamy in Asteraceae specifically evolved by pre-displacement and progenesis of floral development, as well as neoteny of all whorls other than the gynoecium (Porras and Muñoz, 2000). The diversity of floral morphologies within *Jaltomata*, a Solanaceae genus, is due to hypermorphosis and acceleration of some corolla traits (Kostyun et al., 2017). The diversity of Azorean butterfly orchids is also caused by floral heterochronic shifts (Bateman et al., 2014). Recently, Ronse de Craene (2018) emphasized the importance of heterochrony in three developmental processes: phyllotaxis, the development of common stamen-petal primordia and obdiplostemony, linking changes in the growth rate with delayed organ initiation. Heterochronic growth rates of the perianth and style, and early hypanthium elongation, are responsible for the great species diversity within the morphologically homogeneous *Eugenia* genus (Vasconcelos et al., 2018). Finally, heterochronic expression of the *fw2.2* allele, which affects cell division in early fruit development, is responsible for natural variation in tomato fruit size (Cong et al., 2002).

## Studying Heterochronic Mutants to Elucidate Genetic Control of Timing

The study of mutants affected in developmental timing has shed light on genetic pathways controlling morphogenesis and developmental transitions. Heterochrony can be caused by earlier or later activation or repression of these pathways.

*leafy cotyledon (lec)*, *dicer-like1 (dcl1)*, and *extra cotyledon (xtc)* mutants of *Arabidopsis thaliana* represent heterochronic phenotypes that have helped to define seed maturation programs. *lec* mutants produce cotyledons with features of leaf identity (Meinke, 1992), a clear example of homeosis: the replacement of one structure by another. However, it is often difficult to distinguish between homeosis and heterochrony, since homeosis can be the result of both heterochrony and heterotopy (Li and Johnston, 2000; Geuten and Coenen, 2013). During late embryogenesis, *LEC2* promotes seed maturation and represses postembryonic identity (Stone et al., 2008). Like *lec* mutants, *dcl1* mutants show peramorphic phenotypes during embryogenesis, as chloroplast development and seed storage protein gene expression occur earlier than in wild type embryos. *DCL1* is required for biogenesis of microRNAs, which repress seed maturation through the master regulators *LEC2* and *FUSCA3* (Nodine and Bartel, 2010; Willmann et al., 2011). The *xtc1*, *xtc2*, and *altered meristem programming1* (*amp1*) mutants show a homeotic phenotype where the first one or two leaves are transformed into cotyledons (Conway and Poethig, 1997). In these three mutants, the globular to heart transition is delayed, causing an enlarged shoot meristem, which leads to extra organ formation during embryogenesis. This phenotype can be interpreted as hypermorphosis, a type of peramorphosis, since embryo development is extended to a more developed shape (like the peramorphic scenario in **Figure 1B**). However, if vegetative development is chosen as a point of reference, *lec* mutants would represent a case of pre-displacement (peramorphosis), and *xtc1*, *xtc2*, and *amp1* would represent post-displacement (paedomorphosis) in the acquisition of leaf identity. AMP1 is required for miRNA-mediated translational repression on the ER membrane (Li et al., 2013).

Genetic regulation of the juvenile to adult transition, also called vegetative phase change, has been studied using both paedomorphic and peramorphic mutants. Screenings for mutants showing an early adult (peramorphic) phenotype produced alleles of genes related to small RNA biogenesis like *zippy/ago7*, *sgs3*, and *rdr6* (Hunter et al., 2003; Peragine et al., 2004), while mutants with a late adult (paedomorphic) phenotype are due to increased miRNA levels (Gillmor et al., 2014; Xu M. et al., 2016, 2018; Guo et al., 2017). The genetic basis for these phenotypes is explained by altered expression of the closely related microRNAs miR156 and miR157, the main regulators of vegetative phase change that act by repressing *SPL* transcription factors at both transcriptional and translational levels, in a threshold-dependent manner (Wu and Poethig, 2006; Chuck et al., 2007; Poethig, 2013; He et al., 2018). Both *MIR156* and *SPL* gene families are directly regulated by epigenetic marks: *MIR156* is activated by H3K4me3 and H3K4ac, which are promoted by the SWR1-C complex and the chromatin remodeler BRAHMA, and is repressed by H3K27me3, which is promoted by the Polycomb proteins SWINGER and CURLY LEAF and by the chromatin remodeler PICKLE (Xu M. et al., 2016; Xu Y. et al., 2016; Xu M. et al., 2018). *SPL* genes are activated by histone acetylation mediated by the SAGA-like complex and repressed by H2AUb mediated by the Polycomb proteins RING1A and RING1B (Kim et al., 2015; Li et al., 2017). The study of plants with loss- or gain-of function of *SPL* genes has also defined an endogenous flowering pathway in which some *SPL* genes promote the expression of miR172, which in turn promotes flowering by repressing the *APETALA2* family flowering repressors (Wang et al., 2009; Wu et al., 2009; Huijser and Schmid, 2011).

Besides major developmental transitions, organ morphogenesis can also be affected by heterochronic activation of genetic regulators. For instance, early germination of *Brassica rapa* embryos results in organs with mosaics of cotyledon and leaf identity (Fernandez, 1997), and differential temporal expression of the class II of *TEOSINTE BRANCHED1/CYCLOIDEA/PROLIFERATION CELL FACTOR* (*TCP*) genes results in leaves with different size and shape (Efroni et al., 2008).

## Mechanisms Driving Changes in Developmental Timing in Plants (Transcriptional, Metabolic, and Cellular Heterochrony)

**FIGURE 2:**
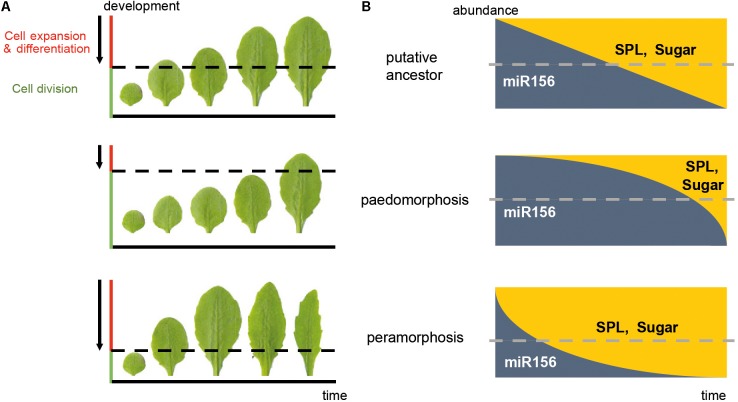
Mechanisms driving heterochrony in plants. **(A)** Cellular heterochrony. The sequence of rosette leaves produced during vegetative development is shown. The phenotype of Arabidopsis Columbia wild type plants is taken as a hypothetical ancestral state; a paedomorphic scenario (neoteny) and a peramorphic scenario (acceleration) are also shown. Arrows represent the direction of the wave of the cell division arrest in a basipetal gradient, where cells below the dotted line are still actively dividing whereas the cells above are expanding and/or differentiating. **(B)** Transcriptional and metabolic heterochrony. A graphic representation of the predicted abundance of the microRNA miR156/7 and its targets, the *SPL* genes, as well as sugar abundance along the time of vegetative development, corresponding to the phenotypes in **(A)**. Dotted lines represent the threshold of miR156/7 and SPL abundance which leads to juvenility (above the threshold) or adulthood (below the threshold). In this case, a delay in the repression of miR156 and activation of SPL expression and sugar production results in paedomorphosis, whereas a precocious decay of miR156 and early activation of SPL expression and sugar production results in peramorphosis. These drawings are simplified representations, and do not reflect the exact abundances in nature. The actual pattern of miR156/SPL abundance is closer to that depicted for “peramorphosis” state.

Transcriptional heterochrony refers to a change in the timing of activation or repression of gene expression, and is often caused by changes in *cis*-regulatory gene regions (Pham et al., 2017). Transcriptional heterochrony in the genetic pathways mentioned above is a common way of producing heterochronic phenotypes. Metabolic control of pathways regulating developmental transitions (referred to here as “metabolic heterochrony”), and temporal control of cell proliferation, cell expansion and cell differentiation (referred to here as “cellular heterochrony”) are other mechanisms driving heterochrony in plants (**Figure 2**). Transcriptional, metabolic and cellular processes are interconnected, so the molecular origin of heterochrony can be due to a combination of mechanisms.

Metabolic heterochrony can be influenced by hormones, sugars, and redox signals (Jia et al., 2017). Differential biosynthesis, transport and perception of hormones such as auxin, jasmonic acid (JA), gibberellin and abscisic acid influence heterochrony by controlling regulators of developmental processes. For instance, mutants in the *AUXIN RESPONSE FACTORS (ARF) ARF3* and *ARF4* delay the adult transition (Fahlgren et al., 2006; Hunter et al., 2006), and auxin homeostasis controls the transition from floral stem cell maintenance to gynoecium formation (Yamaguchi et al., 2017). Exogenous JA can delay the adult transition by postponing the decline of miR156 expression (Beydler et al., 2016), and gibberellin accelerates flowering by releasing *SPL* genes from repression by DELLA proteins (Yu et al., 2012). Nutritional status has been associated with the control of vegetative phase change since the early 20th century (Goebel, 1908). Sugar produced by photosynthesis is necessary for the acquisition of adult traits and is partially responsible for the decrease in miR156 expression in late vegetative development (Yang et al., 2013; Yu et al., 2013; Buendía-Monreal and Gillmor, 2017). *HEXOKINASE1* (*HXK1*) and Trehalose-6-phosphate (T6P) are important for the sugar-mediated repression of miR156, thereby promoting vegetative phase change and flowering (Wahl et al., 2013; Yang et al., 2013).

At the cellular level, organogenesis consists of a sequence of three stages: the establishment of polarity, cell proliferation, and cell expansion (Walcher-Chevillet and Kramer, 2016). The timing of initiation and termination of these stages is crucial for the size and shape of organs, and heterochrony in this sequence results in diversification of organ size and shape (**Figure 2**). The transition from cell proliferation to cell expansion and differentiation requires coordination between the cell cycle and cell growth (Sablowski and Carnier Dornelas, 2014). In leaves of model plants, this transition moves as a basipetal wave of cell cycle arrest that begins at the distal part of the primordium and moves to the base. Cells behind the mitotic arrest front become highly vacuolated and begin to expand (Donnelly et al., 1999; Czesnick and Lenhard, 2015). However, other plant species can show diffuse growth, and acropetal or bidirectional cell cycle arrest gradients (Das Gupta and Nath, 2015). The acquisition of photosynthetic capacity is required for the shift from cell division to cell expansion (Andriankaja et al., 2012). This shift correlates with the role of sugar in promoting the Target of Rapamycin (TOR) pathway and repressing the Sucrose-non-fermenting1-related kinase 1 (SnRK1): TOR and T6P induce cell expansion by promoting macromolecular synthesis, whereas SnRK1 promotes catabolism (Tsai and Gazzarrini, 2014; Sablowski, 2016). Two microRNAs play opposite roles in this cellular shift: miR319 represses the expression of class II TCP factors, which are inhibitors of cell proliferation, whereas miR396 restricts the expression of GROWTH REGULATING FACTORS (GRFs), which delay differentiation (Das Gupta and Nath, 2015; Maugarny-Calés and Laufs, 2018). The transition from an indeterminate shoot apical meristem to a determinate floral meristem also involves temporal regulation of the cellular identity. The timing of *AGAMOUS* activation of *KNUCKLES*, which in turn represses *WUSCHEL*, defines the temporal window of indeterminacy and consequently the size and the number of organs (Sun et al., 2014).

## Conclusion

An understanding of temporal regulation of plant development is necessary to better appreciate the diversity of plant forms that we see in nature, to explain plant morphological evolution, and to manipulate plant architecture for the benefit of agriculture. As outlined above, interrelated transcriptional, metabolic, and cellular mechanisms drive heterochrony in extant species. Further research on these pathways in angiosperms and basal plant lineages should reveal more about the changes in developmental timing that have driven the evolution of development in plants.

## Author Contributions

MB-M conceived and wrote the manuscript. CSG edited and revised the manuscript.

## Conflict of Interest Statement

The authors declare that the research was conducted in the absence of any commercial or financial relationships that could be construed as a potential conflict of interest.
